# Essay on Pneumonia

**Published:** 1859-12

**Authors:** A. Heavenridge

**Affiliations:** Stilesville, Ind.


ARTICLE II.

### ESSAY ON PNEUMONIA.

(Read before the Hendricks County Medical Society.)

BY A. HEAVENRIDGE, M. D., OF STILESVILLE, IND.

*Gentlemen of the Hendricks Co. Medical Society:*

By reason of the legal operations of our organization, it becomes my duty to read a disquisition on some medical subject. I have chosen for that purpose the investigation of that very common, yet very important, disease, familiar to every practitioner of medicine as Pulmonitis or Pneumonia. I am aware that there has been some discrepancy of opinion among nosological writers, as to the precise tissue or tissues involved in pneumonic inflammation; but the more recent writers confine it to the blood-vessels, air cells and inter-cellular tissue, composing the parenchymatous structure of the lungs. It is in that light I wish to be understood as using the term, thereby excluding all the inflammations involving the pleuritic membrane, except the pneumonic inflammations implicating it.

By inviting your attention to a consideration of so common a disease as that of pneumonia, I am aware that I shall create anticipations of the development of something new either in the pathology or treatment of this disease. I only wish, however, to present my views and convictions, that I may know how they compare with the experience of my colaborators in the healing art, at the present stage of medical science. As brevity is the beauty of bad composition, I shall forego an elaborate recitation of all the various theories and views that have, from time to time, been presented by the different authors upon that subject. As the object of my disquisition is brevity, with perspicuity, I shall only consider three varieties, viz., lobular, lobar and double pneumonia.

I shall, likewise, only consider three stages, that of congestion, hepatization and suppuration. There will, also, be only three complications noticed, as the development of pneumonic inflammation under all other circumstances is merely incidental, and consequently does not properly belong to this place. The complications here alluded to are those in which the hepatic function is involved, where the system is in a typhoid condition, and that in which the pleuritic membrane is implicated, constituting the respective types, known as bilious, typhoid and pleuro-pneumonia.

It is in the form of lobular pneumonia that the disease, more generally, appears in infants and children under five and six years old; hence, the name of infantile pneumonia has been given it. In this form of the affection, the disease does not attack a whole lung, or lobe of a lung, at a time, but merely small isolated sections throughout the lung. These small patches of inflammation are diversified in size as well as number. There may only be a lobule involved in the morbid action, or there may be many, thus varying the size of the inflamed sections of lung from that of a millet seed to that of a small orange.

These small points of inflammation may serve as nuclei, from which the morbid action may radiate, uniting one with another until they involve a whole lobe or lung, thus becoming lobar pneumonia. It is in this form of the disease that we are most liable to have pulmonary abscess, formed by the small patches of inflammation running through the various stages of diseased action to suppuration, destroying the parenchyma of the lung involved in the *morbi actio*, the suppurating cavity being surrounded by a plastic wall, thus limiting the abscess, which afterwards collapses, cicatrizes, and leaves the lung sound, though contracted.

I do not wish to be understood as saying that abscess is a necessary result of this variety of pneumonic inflammation.

Why it is that this form of pneumonia is more common in children than in persons of more mature years, must be from the fact, that the pulmonary tissue of children is more susceptible of inflammations in general than that of older persons; and also, that the disease spreads more slowly through the tissue, and thus enables us to detect it before the inflammatory process has radiated throughout the entire lung or lobe.

In respect to lobar pneumonia, I am not aware that there is anything peculiar about this form of the complaint except that it attacks an entire lobe or lung at once; and it is likewise in this form that we have the affection occurring most frequently. In double pneumonia, we have but to consider the inflammation of both lungs, or the entire breathing apparatus of man. In this form of the complaint, the danger is greatly augmented; consequently, the prognosis is rendered more dubious than in either of the other varieties. While upon this point I will say, that this form of the disease seldom passes beyond the stage of hepatization, the patient sinking before the stage of congestion is fairly passed.

In passing to the consideration of the different stages through which the different varieties of pulmonary inflammation run, when not prevented by treatment, or the voluntary resolution of the diseased action, I am aware that I am entering upon the investigation of a subject that admits of mooting to a degree far beyond any information of the physician, or good to the afflicted. In the investigation of the first, or congestive stage of this affection, it is proper to observe, that the inflammation is preceded here, as elsewhere, by irritation of the parts and by a determination of blood thereto, giving rise to a fulness of the capillary blood-vessels of the lungs. Hence, it has been claimed by some pathological writers that this forms one stage of the complaint. But, as all cases of irritation and congestion of the lungs do not pass into inflammation, it forms no part of this disease, but merely remains as congestion, and requires to be treated as such.

We have, then, in the true congestion of inflammation, not only local hyperæmia of the capillaries of the lungs, but an infiltration of the inter-cellular tissue and air cells with the liquor sanguinis, thus evolving that well known and truly diagnostic physical symptom, *crepitation*, which is produced by filling the air cells with fluid, at the same time infiltrating the cellular tissue, and thus compressing the air cells, rendering the ingress of air difficult, labored and rattling: hence the crepitation.

In this stage of the inflammation, the healthy respiratory murmur gradually or rapidly gives place to the crepitation, according as the atttack has been mild or severe. In the lobular form, the healthy respiratory murmur may be heard intermingling with the crepitant rale; and it is chiefly upon this diagnostic sign that we have to depend for our information as to which variety of the complaint may be present. The rational symptoms of this stage are pain in the chest, cough, hurried respiration, a hot and dry skin, a full, firm and frequent pulse, with a white or yellowish-white coat upon the tongue, accompanied with red expectoration; the bowels are generally constipated, though by no means invariably so. The urinary organs are, for the most part, inactive; the urine, when voided, bearing evidence of febrile excitement in the system. By far the most valuable of these symptoms is the expectoration of the characteristic sputa. But even this will not enable us to say conclusively that pulmonitis is present, from the fact, that the same kind of sputa—at least, apparently the same—may be produced from other causes than pneumonic inflammation of the lungs.

But while we are left in doubt as to the real nature of the disease by a mere reference to the rational symptoms, those doubts no longer exist when we come to consider the physical symptoms. And even here percussion affords little or no evidence of disease, as the lungs still remain, if not entirely as resonant as in health, so nearly so that it is with difficulty that the difference can be detected. But when we come to auscultate the lungs, we are no longer left in doubt as to the real nature of the affection. Crepitation is heard, and we say the patient has pneumonia, in a greater or less degree, according to the extent of the crepitation.

In the second or hepatized stage of this complaint, we find the inter-cellular tissue and air cells so firmly impacted with the infiltrating fluid that the air is no longer permitted to enter the air cells. Upon the accession of this stage, the breathing becomes more frequent, the face and extremities of a dingy hue, in consequence of the presence of an excess of carbon in the blood. The tongue becomes brown; pulse frequent and small. On auscultating the lungs, no sound is heard except bronchial respiration; and upon the patient speaking, bronchophony may be heard, in consequence of the consolidation of the lung, affording a more compact medium through which the voice is more readily transmitted to the ear of the auscultator.

It is said by pathological writers that there are two kinds of hepatization,—the *red* and *gray;* but I am of opinion that they are only different stages of the affection,—the red being primary and the gray being secondary, or the gray being the inception of the third or suppurative stage. Should the disease take a favorable turn during the first stage, it passes immediately into the third. Suppuration, with absorption of the effused fluids, commences; that contained within the air cells being changed into pus and expectorated, while absorption carries away that contained within the inter-lobular tissue, and thus convalescence is established.

Should the second stage terminate without abscess, the same thing is effected. When the extravasated fluid fails to be absorbed, abscess is an unavoidable consequence. The degeneration into pus of the effused plastic lymph produces softening and disintegration of the parenchymatous structure of the lungs, and, as a matter of course, abscess is the legitimate result. Should this softening be extensive, the patient sinks, and when the lung is seen, it presents a dark, motley or variegated appearance: and this is said to be gray hepatization. I do not think it possible for a lung, or any part of it, affected with what is known as gray hepatization, ever to return to health; the vitality must inevitably be too far spent for the parts to recover.

In reference to the complications of pneumonic inflammation of the lungs, I will first notice that in which the liver and its functions are involved. It is such a constant concomitant of this form of pulmonary disease that it amounts to almost a necessary part of the disease. The reason why this complication is so persistently present is obvious, when we consider the effect that capillary turgescence of the lungs has upon the liver, producing hyperæmia of the right side of the heart, and consequently of the entire venous circulation, but more especially of the capillaries of the liver. The anatomical structure of the parts is such, that the blood passes directly from the liver to the lungs.

It follows that this passive congestion of the venous capillaries must induce derangement in the hepatic function.

Pneumonia of the lungs has such a direct influence upon the liver, that I have not unfrequently known hepatitis to be superadded to the pulmonary inflammation. In this complication we have all the symptoms of biliousness added to those of pulmonitis, such as a yellowish coat upon the tongue, a sallow complexion of the skin, with icterus of the conjunctiva, a straw colored and diminished urinary secretion.

In passing to the investigation of the typhoid complication of this disease, we are met by the reflection, that we have to do with a more grave commingling of diseased action than in any form of the disease that has passed under review. In this form of the complaint, we have, in addition to the pulmonary phlegmasia, a loss of the vital forces of the system. An aplastic condition of the blood obtains, with loss of susceptibility and vital tonicity, of all the tissues of the body. Such a state of the system is made manifest by the slow and unsteady action of the muscular movements, coupled with a dingy expression of the countenance, a frequent, soft and easily compressed pulse, with frequent mental aberrations, and, finally, delirium.

Inflammation occurring in such a state of the system must necessarily be more obscure, and less distinctly marked than when occurring in a vigorous and healthy condition of the organism. The inflammation is more disposed to become diffuse. The blood having lost, to a greater extent, its plastic properties, the inflammatory process is not circumscribed by a plastic exudation, so necessary to prevent its spread. Thus, we seldom have lobular pneumonia in typhoid conditions of the system.

The sputa in this condition is more copious, looking as though it was blood and mucus that had been squeezed through the coats of the blood-vessels and mucous membranes into the air cells. The crepitation, likewise, partakes more of the mucous rale.

Further on in the disease, we have developed all the symptoms of typhoid fever, such as a frequent and feeble pulse, continued delirium, sordes and a dry tongue, with tympanitis. The local phenomena are a flat sound, on percussion over the diseased portion of the pulmonary tissue, with bronchial respiration and bronchophony on auscultation.

Except the increased debility, we have little else than a mere change in the physical symptoms of the second stage of this complaint to constitute the third. The physical symptoms are diminished dulness, with a return of the mucous rale, or rather the establishment of the mucous râle.

Should the inflammation be extensive, this stage of the complaint is extremely dangerous, from the copious suppuration exhausting the remaining strength of the patient, and its filling the air cells with pus, and thus preventing the aeration of the blood.

The third and remaining complication of pneumonia that I propose to consider is extensive inflammation of the pleuritic membrane, which, in fact, is nothing more than a super-addition of pleuritis to pulmonitis, constituting pleuro-pneumonia.

The causes that produce inflammation of the lungs are (like those of all other diseases that are not dependent upon a specific virus or poison) various, and many times not very apparent or easily detected; as mechanical violence, the inhalation of noxious vapors, and catarrhal inflammation of the mucous membrane of the air passages propagated to the substance of the lungs; but by far the most prolific cause seems to be an epidemical influence. What this epidemical influence consists in I am not aware of; the circumstances and materials necessary to evolve such influence are equally obscure; but certain it is that such epidemical influence many times prevails. So observable is this that each epidemic almost always has something peculiar to itself, either in its symptomatology, pathology or therapeutical indications.

Gentlemen, as the therapeutical management of a disease is the part which most interests the physician, and that upon which the welfare and happiness of the afflicted more immediately depends, I hope you will pardon me should I presume upon your patience in giving rather a minute statement of what I consider the best means of filling the therapeutical indications in the disease under consideration.

Before proceeding, however, to give my views upon the treatment of pulmonary inflammation, I propose to look for a few moments at the teachings of some of the writers of the text books. From the teachings of the books in reference to the treatment of this disease, the novitiate in the *ars medendi* is irresistibly led to the conclusion that all he has to do to cure his pneumonic patients is to bleed them to syncope, and if reaction is well marked, they are to be bled down again; and even a third and fourth full bleeding is recommended should the inflammation not yield. After the physician has bled the patient as long as he can lose blood and live, he must then, according to some of the older therapeutical writers, administer tartarized antimony, in small and increasing quantities, until it would no longer act as an emetic, continuing it until the stomach would tolerate it in enormous quantities; and it is asserted by the advocates of such treatment that the greater tolerance shown by the system for the remedy, the more effectual it is in subduing the inflammation. Should the patient and disease both withstand the lancet and antimony, as above indicated, mercury was then to be tried; and nothing short of mercurialization of the system was considered sufficient. The object of the mercury, I suppose, was to dissolve the plasticity of the remaining blood, and leave nothing to propagate the inflammation.

The more recent writers on practical medicine recommend a less heroic employment of such spoliative measures; nevertheless, they rely on them as their *curative* means. Venesection is employed for the double purpose of diminishing the quantity of the circulating fluid, and thus relieving the labor of the inflamed organs, and also to cut the inflammation short. Antimony was administered with reference to its powerful sedative and absorbing influence upon the system. Calomel was used to dissolve the morbid plasticity of the blood, thus incapacitating it to sustain a phlogistic condition of the system.

The above means, with the addition of opium, blisters, expectorants, and, in the latter stages, stimulants, constitute the treatment of the books. I do not wish to be understood as advocating the condemnation of the foregoing plan of medication in its more moderate and judicious administration: so far from it, I have not only seen others practice it with commendable success, but have done so myself. I am of opinion, however, that at the present time such practice falls short of the present standard of medical science. Such treatment entails upon the patient a tedious convalescence, requiring weeks and sometimes months to regain complete health. The employment of such spoliative measures implies a reduction of the vital forces of the animal economy to a point below which the inflammatory process cannot exist, which must be wholly inapplicable to those cases occurring in a typhoid condition of system.

In regard to the benefit to be derived from venesection, in acute inflammation of the lungs, I am of opinion that in subjects of a plethoric condition of system there is no single agent that the physician can employ that will do so much in facilitating the cure as the early premonition of a full bleeding. But with our present means of controlling arterial action, I am convinced that the cases must be very rare in which a second blood-letting is indicated; indeed, the number requiring venesection at all is small in proportion to the entire number of cases of lung inflammation. It is a remedial agent, the employment of which is fraught with extreme danger in those cases in which the typhoid character is well marked.

As visionary and chimerical as it may seem, I am of the opinion that after the premonition of blood-letting, in those cases requiring it, and cathartics in case of constipation of the bowels, there are no agents that the physician can employ that will facilitate the resolution of the inflammation so much as a liberal use of quinine and opium. In those cases of a less phlogistic type, with a lax or unconstipated condition of the bowels, I would exhibit the quinine and opium, in full doses, until the constitutional effect of both remedies were produced. At the same time, I would bring the arterial circulation within the point of active secretion by means of veratrum viride.

This condition of the system I should aim to sustain until I had reason to believe that the inflammatory process was stopped, which generally takes place in from two to five days. The amount necessary to be given at once would vary according to the age, constitution and susceptibility of the patient;—from two to three grains of the pulvis opii, with from four to five of the salts of quinia, repeated every three, four or five hours, will generally be found sufficient in most cases of adults; though should this amount be found insufficient to completely quiet the cough and establish active diaphoresis, more should be given.

I am aware that the argument will be here adduced that the exhibition of such large and repeated doses of opium will dry up the secretions. In reply, I would say that is just what I would expect to do so far as the bronchial secretions are concerned, which is evidenced by the diminished expectoration; but at the same time I would expect to powerfully stimulate the sudorific exhalation.

During the administration of these remedies, the bowels should be acted upon as often as necessary. It will generally be found that after the system is completly controlled by the quinine and opium, that the arterial sedative is no longer required to diminish the arterial circulation.

I will digress sufficiently here to say, that the most prolific cause of failure of this plan of medication will generally be found in a deficient exhibition of the remedies. The *rationale* of the foregoing treatment is obvious on a moment's reflection. The opium allays the cough and irritation; hence, the determination of blood to the lungs ceases. The cough—the mechanical means by which the blood is forced through the pulmonary capillaries into the inter-cellular tissue and air cells—being quieted, we no longer have bloody expectoration. The opium further acts in connection with the quinine in equalizing the circulation, and in producing capillary turgescence of the blood capillaries of the skin. And thus we have diaphoresis, with the excess of blood as far removed from the seat of diseased action as is possible, yet remaining and sustaining the vital forces of the system. Should the opium not be administered so as to completly quiet the cough, the practitioner will fail of accomplishing his object, that of resolving the inflammation.

By thus administering these agents, the circulation is equalized, irritation allayed, pulmonic determination suspended, and consequently capillary tension removed; and the effused fluids no longer meeting with resistance, pass back into the blood-vessels, by reason of the laws of endosmosis. The plastic fluid that may have been poured into the air cells is expectorated, and the lung passes, by an easy and rapid transit, from a state of active pulmonic inflammation to health, and that, too, without the patient passing through a tedious convalescence of weeks, as when such heroic depletory measures are employed as those above alluded to.

In uncomplicated cases of pneumonia, little else will be found necessary than to premise a full bleeding. Should the patient be plethoric, clear the bowels with an active cathartic, and then administer the quinine and opium in such quantities as to ensure their quieting and sudorific effects; controlling, at the same time, the arterial circulation by means of veratrum viride or digitalis. Should the inflammation be found obstinate in yielding, a blister will be useful in the latter part of the treatment. The system should be controlled by anodynes and tonics until convalescence commences, and then they should be gradually left off. Expectorants seldom afford much benefit in the treatment of the first stage of this complaint, except convalescence should be tedious.

In regard to the treatment of the stage of hepatization, I will only add, that blisters will be found extremely useful, after the depletory and sedative measures have been thoroughly premised. During the stage of suppuration, tonics, stimulants and expectorants are the remedies upon which we must rely to sustain the patient through the exhausting process. As tonics, the various preparations of Peruvian bark are useful, and perhaps none more so than the salts of quinia.

As a diffusible stimulant and expectorant, I have found no preparation so useful as a combination of carbonate of ammonia with a decoction of senega. Sanguinaria Canadensis, in combination with an opiate, I have found very useful as an expectorant in this stage of the disease. Wine and animal broths should be liberally administered, to sustain the patient during the exhaustion of suppuration.

In treating the complications of pneumonia it will be observed, that to make the treatment meet every indication of that very common form of the disease called bilious pneumonia, the administration of a sufficient quantity of mercurial to stimulate the hepatic function will be all that is necessary, in addition to the plan of medication just indicated. This may be done by a mercurial pill, or a few grains of calomel at night.

In vigorous subjects with constipation of the bowels, I have found a powder, composed of calomel and podophilline, very useful in cleaning the bowels, stimulating the hepatic function, and equalizing the circulation.

In the typhoid complication of pneumonia, I am convinced that blood-letting is an extremely dangerous practice. The amount of local benefit derived from general bleeding will not compensate for the injury done the system while laboring under a disease tending so rapidly to debility, the progress of which can be so little influenced by medication. I should, therefore, depend upon anodynes, alteratives, tonics, blisters and sedatives for a resolution of the local inflammation. Mercury should be given with reference to its moderate constitutional impression, not only as being the best means of managing the fever due to the typhoid state of system, but by its absorbing effect—the best agent we have to resolve the pulmonic inflammation in such a condition of system. In the hepatized condition of the lungs, in typhoid pneumonia, blisters will be found to exert an influence of unusual benefit in discussing the inflammation. As suppuration comes on, tonics, wine, diffusible stimuli and stimulating expectorants should be resorted to; and I have found the administration of chlorate of potassa of benefit in this stage, where there is embarrassment of respiration with flagging of the vital powers, in consequence of an excess of carbon in the blood.

In treating that form of disease in which an extensive inflammation of the pleuritic membrane gives rise to that complication known as pleuro-pneumonia, in addition to the treatment indicated for simple pneumonia, it will only be necessary to add, that after all the depletory measures that are necessary have been premised, and the acuteness of the attack has been subdued, blisters over the seat of the pleuritic inflammation will generally be found beneficial.

Should the pleuritic inflammation become chronic, and persist after the lungs are relieved, counter-irritation, by means of croton oil blisters or tartar emetic ointment, with the internal administration of iodide of potassium, muriate of ammonia, or mercury, until it affects the system moderately, will generally be found sufficient to complete the cure.

The infantile form of pneumonia—or, rather, pneumonia occurring in infants—will require some variation in treatment, in consequence of the extreme susceptibility to opium manifested during infancy. That remedy will have to be exhibited with caution. But it will almost always be found that they will bear a sufficiency of the medicine to control the cough; and as that is the *sine qua non* to the resolution of the inflammation, it should be given with reference to that result. This, with some other trifling precautionary measures, which the wisdom of the practitioner will suggest, will be sufficient for the management of the complaint as occurring in infants.

